# Statistical density modification using local pattern matching

**DOI:** 10.1107/S0907444903015142

**Published:** 2003-09-19

**Authors:** Thomas C. Terwilliger

**Affiliations:** a Mail Stop M888, Los Alamos National Laboratory, Los Alamos, NM 87545, USA

**Keywords:** density modification, pattern matching

## Abstract

A method for improving crystallographic phases is presented that is based on the preferential occurrence of certain local patterns of electron density in macromolecular electron-density maps. The method focuses on the relationship between the value of electron density at a point in the map and the pattern of density surrounding this point. Patterns of density that can be superimposed by rotation about the central point are considered equivalent. Standard templates are created from experimental or model electron-density maps by clustering and averaging local patterns of electron density. The clustering is based on correlation coefficients after rotation to maximize the correlation. Experimental or model maps are also used to create histograms relating the value of electron density at the central point to the correlation coefficient of the density surrounding this point with each member of the set of standard patterns. These histograms are then used to estimate the electron density at each point in a new experimental electron-density map using the pattern of electron density at points surrounding that point and the correlation coefficient of this density to each of the set of standard templates, again after rotation to maximize the correlation. The method is strengthened by excluding any information from the point in question from both the templates and the local pattern of density in the calculation. A function based on the origin of the Patterson function is used to remove information about the electron density at the point in question from nearby electron density. This allows an estimation of the electron density at each point in a map, using only information from other points in the process. The resulting estimates of electron density are shown to have errors that are nearly independent of the errors in the original map using model data and templates calculated at a resolution of 2.6 Å. Owing to this independence of errors, information from the new map can be combined in a simple fashion with information from the original map to create an improved map. An iterative phase-improvement process using this approach and other applications of the image-reconstruction method are described and applied to experimental data at resolutions ranging from 2.4 to 2.8 Å.

## Abstract 2

Statistical density modification can make use of local patterns of density found in protein structures to improve crystallographic phases.

## 1. Introduction

Electron-density maps corresponding to macromolecules such as proteins have features that differ in fundamental ways from those found in maps calculated with random phases. These differences have been used in many ways, ranging from improving the accuracy of crystallographic phases to evaluating the quality of electron-density maps. For example, maps corresponding to proteins often have large regions of relatively featureless solvent and large regions containing of polypeptide chains, while a map calculated with random phases has similar fluctuations in density everywhere (Bricogne, 1974 ▶). This observation is the basis of the powerful solvent-flattening approach (Bricogne, 1974 ▶; Wang, 1985 ▶) as well as methods for evaluating the quality of macromolecular electron-density maps (*e.g.* Terwilliger & Berendzen, 1999 ▶). Similarly, the presence of non-crystallographic symmetry in macromolecular electron-density maps has been useful in phase improvement (Bricogne, 1974 ▶; Rossmann, 1972 ▶; Kleywegt & Read, 1997 ▶). Additionally, maps corresponding to macromolecules can be interpreted in terms of atomic models, providing a powerful basis for map-quality evaluation and improvement (Agarwal & Isaacs, 1977 ▶; Lunin & Urzhumtsev, 1984 ▶; Lamzin & Wilson, 1993 ▶; Perrakis *et al.*, 1997 ▶, 1999 ▶, 2001 ▶; Morris *et al.*, 2002 ▶). On a statistical level, the density in the protein region of a macromolecular electron-density map has a distribution that is very different to that in a map calculated with random phases. This has been extensively used in histogram matching and related methods for phase improvement (Harrison, 1988 ▶; Lunin, 1988 ▶; Zhang & Main, 1990 ▶; Zhang *et al.*, 1997 ▶; Goldstein & Zhang, 1998 ▶; Nieh & Zhang, 1999 ▶; Cowtan, 1999 ▶).

In this work, the focus is on local patterns of density that are common in macromolecular protein structures. Macromolecules are built from small regular repeated units and the packing of these units is highly constrained owing to van der Waals interactions. Owing to the regularity of macromolecules on a local scale, their electron-density maps have local features that are distinctive and very different from those of maps calculated from random phases (Lunin, 2000 ▶; Urzhumtsev *et al.*, 2000 ▶; Main & Wilson, 2000 ▶; Wilson & Main, 2000 ▶; Colovos *et al.*, 2000 ▶). This property has been used to evaluate the quality of electron-density maps and to improve phases at low resolution. Lunin (2000 ▶), Urzhumtsev *et al.* (2000 ▶), Main & Wilson (2000 ▶) and Wilson & Main (2000 ▶) use histogram and wavelet analysis to improve electron density in low-resolution maps by requiring the wavelet coefficients to be similar to those of model structures. Colovos *et al.* (2000 ▶) analyze the local features of high- and medium-resolution electron-density maps and compare them with those of model maps to evaluate the quality of the maps and suggest that their approaches may also be useful for phase improvement.

We recently developed a method for density modification that consisted of the identification of the locations of helical or other highly regular features in an electron-density map, followed by statistical density modification using an idealized version of this density as the ‘expected’ electron density nearby (Terwilliger, 2001 ▶). This method was shown to yield some phase improvement, but suffered the serious disadvantage that after an initial cycle the features that were initially identified became greatly accentuated and few new features could be found. We suspect that this is a consequence of the inherent feedback in the method, where a feature in the original electron density that partially matches a helical template is restrained to look like this template, making it an even better match for the template in the next round (even if the true density in the region is not helical). We have therefore developed a very different approach to using the information inherent in local features of an electron-density map which does not have this feedback and which therefore might have substantially improved capability for phase improvement.

Here, we show that the local patterns of density surrounding any point in a map can be used to estimate the electron density at that point. This observation makes it possible to begin with an electron-density map with errors, to obtain a new estimate of the density at each point in the map without using the density at that point and thereby to construct a new estimate of electron density that has errors which are nearly uncorrelated with the errors in the original map. This recovered ‘image’ of the electron density has many uses, including phase improvement and evaluation of map quality.

## 2. Methods

### 2.1. Estimation of electron density from local patterns in a map

The central approach of this work is to use the density surrounding each point in a map to construct a new estimate of electron density at that point. There are three overall steps. The first two create templates and evaluate statistics of these templates using data from experimental or model maps, with and without additional errors. The third applies these results to other maps. In the applications described here, we have used density-modified experimental maps obtained from MAD or SAD data at a resolution of 2.6 Å to create the templates and histograms, but a similar procedure could be carried out using either experimental or model maps at any resolution. In the first step, *N* templates of averaged density are created. These templates were based on the local density in a density-modified experimental protein electron-density map and are grouped by correlation coefficient. Secondly, the relationship between the density at point *x* and the template which has the highest correlation with the density near *x* is tabulated using additional density-modified experimental electron-density maps. Finally, the method is applied to other experimental maps. The density near each point *x* in a map is used to construct a new estimate of the density at *x*. In this process, the local density is corrected in a way that removes the information about the density at *x* from all its neighbors.

### 2.2. Removal of information about density at *x* from local density

In our approach, the goal is to obtain an estimate of the value of the electron density at a point *x* in the unit cell in such a way that the new estimate has errors that are not correlated with the errors in the original electron-density map at *x*. To do this, the method uses information from the electron density at points surrounding the point *x* in obtaining a new estimate of the value of the electron density at *x*. One way to remove the information about the electron density at *x* would simply be to consider the electron density in a spherical shell around the point *x*. If the inner radius of the shell were large enough, then the values of electron density inside the shell would be relatively uncorrelated with the electron density at *x*. The choice of an inner radius, however, is not obvious because the electron-density map is a Fourier sum of terms with widely varying spatial frequencies. Consequently, there is significant correlation between values of electron density at point *x* with points even as far away as the resolution of the map. Additionally, it is disadvantageous to exclude all density close to *x* in the calculations because the patterns to be considered are very local.

An alternative method is to create a local density function for points near *x* that has values that are similar to the electron density near *x*, but that are adjusted in such a way that the values are uncorrelated with the electron density at *x*. This modified local density *g*_*x*_(Δ*x*) will depend on the coordinate difference Δ*x* between each point near *x* and *x*. The function *g*_*x*_(Δ*x*) is a function of both *x* and Δ*x* and therefore must be calculated separately for each point *x* and offset Δ*x* in the map. We would like the value of the function *g*_*x*_(Δ*x*) to be generally similar to the value of the electron density at *x* + Δ*x*, which we will represent by ρ(*x* + Δ*x*). As Δ*x* is increased, we would like *g*_*x*_(Δ*x*) to become very close to ρ(*x* + Δ*x*). That is, we would like
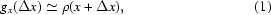

               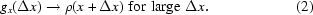
We would also like the function *g*_*x*_(Δ*x*) to be uncorrelated everywhere with the value of the electron density at *x*, given by ρ(*x*). The function *g*_*x*_(Δ*x*) gives modified values of the density at *x* + Δ*x*. We would like to be able to say that if we compare the modified density at *x* + Δ*x* [given by *g*_*x*_(Δ*x*)] with the density at *x* [given by ρ(*x*)], these quantities should be unrelated [that is, *g*_*x*_(Δ*x*) does not contain information about the value of ρ(*x*)]. One way to specify this is to require that for any offset Δ*x*, if we go through the entire map and calculate *g*_*x*_(Δ*x*) for each point *x*, then *g*_*x*_(Δ*x*) and ρ(*x*) are to be uncorrelated,
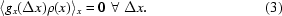
A final desirable property of *g*_*x*_(Δ*x*) for the current purpose is to have its value at Δ*x* = 0 be equal to the mean value of *g*_*x*_(Δ*x*) for nearby points Δ*x*. The reason this is desirable is that we would like to compare local patterns to a template based on the correlation of densities and have no contribution from the mean value of local density. Setting the value of *g*_*x*_(Δ*x*) to any fixed value (*e.g.* zero) at Δ*x* = 0 would introduce a contribution that comes from the mean value of local density ρ(*x*) to the correlation between *g*_*x*_(Δ*x*) and a template. A way to remove information about the mean value of local density is to specify the requirement that
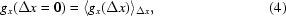
where all values of Δ*x* in the region to be used later in calculations of correlations of densities are considered in the averaging.

A function *g*_*x*_(Δ*x*) that has all these properties is
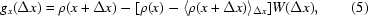
where the weighting function *W*(Δ*x*) is given by
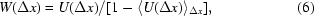
and where the function *U*(Δ*x*) is the normalized value of the Patterson function near the origin, calculated from the electron-density map itself using the relation 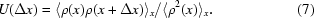
In essence, *g*_*x*_(Δ*x*) is equal to the value of the electron density at *x* + Δ*x*, after correction for the difference between ρ(*x*), the value of the electron density at *x*, and 〈ρ(*x* + Δ*x*)〉_Δ*x*_, the mean of nearby values, all using the weighting function *W*(Δ*x*). It can be verified by substitution that both (3) or (4) are satisfied by this function. Additionally, (1) and (2) are satisfied because the normalized rotationally averaged Patterson function is normally quite small everywhere except near the origin and normally becomes very small for points far from the origin.

### 2.3. Local pattern identification

The first step in the procedure for density modification by pattern matching is to obtain templates that correspond to common patterns of local electron density. These templates are generated using the local electron density near each point *x* in density-modified experimental electron-density maps, modified to remove information from the central point *x*, as described in the previous section. The maps can be calculated at any resolution, but a set of templates is normally associated with a particular resolution (typically *d*_min_ = 2.6 Å). The approach used here to obtain templates is hierarchical. First, three separate sets of *N*_max_ (typically 40) templates are generated using only points in an electron-density map that have low, medium or high electron density. A subset (typically 40) of these templates that have low mutual correlation is then selected. Finally, an even smaller subset of *N*_final_ (typically 20) templates is chosen from this group in order to maximize the predictive power of the templates while maintaining a fixed number of total templates.

To generate a set of templates, each grid point in an electron-density map is considered, one at a time, only including points that are associated with either low (ρ < 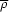 − 0.8σ), medium (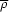 − 0.2σ < ρ < 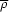 + 0.2σ) or high electron density (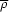 + 1.5σ < ρ), where 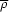 and σ are the mean and standard deviation of the map, depending on the set of templates to be created. For each appropriate grid point (*x*), the modified local electron density *g*_*x*_(Δ*x*) is calculated for all neighboring points within a radius *r*_max_ (typically, *r*_max_ = 2 Å when *d*_min_ = 2.6 Å). This modified electron density is compared with all existing templates using the correlation coefficient of density in the template with the modified local density as a measure of similarity. The grid used is normally the same grid as is used for all FFT, NCS-averaging and other density calculations and is typically between 1/6 and 1/4 of the resolution of the map. The number of points typically used in a template is approximately 100. For each existing template, *N*_rot_ different rotations of the template are considered so as to attempt to match the modified local density in any orientation and the highest correlation coefficient of the match for all rotations of the template is noted. In the examples considered here, we use a total of *N*_rot_ = 158 rotations to sample the possible three-dimensional rotations of an object with a rotation of about 50° relating neighboring orientations. If the correlation coefficient of the local modified electron density at this point *x* with an existing template *k* is greater than CC_min_ (typically, CC_min_ = 0.85), then the local modified density at this point is included in the definition of template *k* by rotating the density to match the current template *k* and including the rotated local modified density in the average density for this template. If the local modified electron density does not have a correlation with any existing template greater than CC_min_, then the local modified density is used to start a new template. Once *N*_max_ templates have been created (typically, *N*_max_ = 40), then the local modified density at each subsequent point is included in whichever template it matches most closely.

By repeating the generation of templates using points in the electron-density map that have low, medium and high density, a relatively diverse set of templates is created. Next, a subset (typically 1/3) of these is chosen based on mutual correlation coefficients in order to obtain a set of templates with the minimum possible similarity to each other. To do this, the correlation coefficients of all pairs of templates are calculated and the template with the highest correlation to another template is eliminated. The process is repeated until the desired number of templates is obtained. The final selection of templates based on predictive power is carried out after analyzing the statistics associated with each of the *N*_max_ templates obtained at this stage, as described in a later section.

### 2.4. Statistics of local patterns: general approach

The second overall step in this process is to identify the relationships between the correlation of each template with local modified density in a map and the value of the electron density at *x*. This is peformed for experimental maps both with and without added errors. There are many possible ways to describe these relationships, but a simple approach used here is to break it down into two parts.

The first part consists of an examination of the statistics of high-quality experimental maps. We have found that the electron density at a point *x* in a map is quite strongly dependent on the two templates *k* and *l* that have the highest (*k*) and next-highest (*l*) correlation coefficients with the local modified density at *x*. That is, for electron-density maps of proteins, the probability distribution *p*(ρ|*k*, *l*) can be very informative about the electron density ρ at *x*.

The second part is to consider the relationship between maps with and without added errors. The approach is to begin with the observed correlation coefficients of all the templates at a point *x* to a map that contains errors and then to use these in a calculation of the probability that a particular pair of templates *k* and *l* would have the highest two correlation coefficients in the corresponding high-quality map. In this case, the statistics of density for the high-quality maps *p*(ρ|*k*, *l*) obtained above can then be applied.

To carry this process out, a second set of probabilities are needed. These are the probabilities *p*(CC_*k*_|CC_obs,*k*_) that the correlation coefficient for template *k* to a point *x* in a high-quality map would have the value CC_*k*_, given the observation that this template has a correlation coefficient of CC_obs,*k*_ to the same point in a map with additional errors. To account for differing levels of error in the experimental map, these probabilities are tabulated as a function of the overall figure of merit of the map with errors.

To apply these probability distributions to data near the point *x* in a new (‘observed’) electron-density map, the correlation coefficient of each template *k* to the local modified density near *x* is first determined (once again, after trying many rotations and choosing the one for each template that maximizes the correlation coefficient). This set of correlation coefficients {CC_obs_} and the two probability distributions *p*(ρ|*k*, *l*) and *p*(CC_*k*_CC_obs,*k*_) can then be combined as follows to obtain an estimate of the electron density ρ at *x* in a high-quality version of the same map.

If we somehow knew which two templates *k* and *l* have the highest correlation coefficients to the local modified density near *x* in a high-quality version of the new ‘observed’ map, then we could use our probability distribution *p*(ρ|*k*, *l*) directly to estimate the probability distribution for ρ. We do not know the identity of *k* and *l*, but suppose instead that we had probabilities, *p*(*k*, *l*|{CC_obs_}), for each possible pair *k* and *l* based on the correlation coefficients observed for the ‘observed’ map. Combining these, we could write that
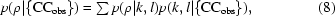
where the sum is over all possible pairs of templates *k* and *l*. An estimate of the electron density at *x* can then be obtained from the weighted mean
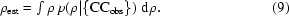
The probability, *p*(*k*, *l*|{CC_obs_}), that the pair *k* and *l* have the highest correlation coefficients to the local modified density near *x* in a high-quality version of the ‘observed’ map can in turn be estimated from the observed correlation coefficients of all the templates to this map, {CC_obs_}, in several steps. We separate the probability into two parts, one for the probability that template *k* has the highest correlation and one for the probability that template *l* has the next highest, given that template *k* has the highest correlation, 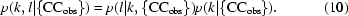
We can now estimate the probability that template *k* has the highest correlation with the (non-existent) high-quality version of the ‘observed’ map. We will integrate over all possible values of CC_*k*_, the correlation of template *k* with the high-quality map. For each value of CC_*k*_, we will calculate the probability that this is indeed the value of the correlation of template *k*, given by *p*(CC_*k*_) = *p*(CC_*k*_|CC_obs,*k*_), and the probability that all other templates have a correlation coefficient less than CC_*k*_,
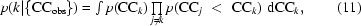
where the integral is over all values of CC_*k*_. The probability that template *l* has the next-highest correlation is given by
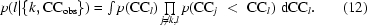

            

### 2.5. Statistics of local patterns: tabulating histograms

An important part of this step consists of generating histograms of values for the electron density at *x* as a function of the correlation coefficients of the *N*_max_ templates with the local modified density at *x*. Each of the *N*_max_ templates is compared with the modified local density at all points in a set of high-quality maps. At each point *x*, the two templates *k* and *l* that have the highest and next-highest correlation coefficients, respectively, with the local modified density at *x* are identified (after rotation to maximize this value). The value of the (unmodified) electron density ρ(*x*) is then tabulated as a function of *k* and *l*. These histograms are then normalized to yield an estimate of the probability distribution, *p*(ρ|*k*, *l*).

The second part of this step is to obtain probability distributions, *p*(CC_*k*_|CC_obs,*k*_), relating the correlation coefficient value, CC_obs,*k*_, observed for a particular template at a point *x* in a map that contains added errors to the correlation coefficient, CC_*k*_, that would be observed for the identical template at the identical point *x* in the corresponding map without any added errors. These probability distributions are calculated by using paired sets of high-quality experimental maps with and without added errors. At each point in a map, the correlation coefficient of each template *k* to the map without added errors, CC_*k*_, and the correlation to the map with added errors, CC_obs,*k*_, are noted. Normalization of the resulting histograms leads to an estimate of the probability, *p*(CC_*k*_|CC_obs,*k*_), that CC_*k*_ is the correlation to the map without added errors if the value CC_obs,*k*_ is observed in the map with added errors. This calculation is repeated for maps with varying levels of additional errors by creating simulated phase sets with Gaussian distributions of phase errors with varying overall values of the cosine of phase error, 〈cosΔϕ〉, ranging typically from 0.5 to 0.8. In application to new ‘observed’ map, the probability distribution obtained using data with added phase errors with a mean cosine 〈cosΔϕ〉 similar to the figure of merit of the experimental map is used.

### 2.6. Selection of templates based on predictive power

The final selection of *N*_final_ templates is based on predictive power. A subset of *N*_final_ templates is selected from the *N*_max_ templates obtained earlier using high-quality electron-density maps. The subset is selected to maximize the correlation between the electron density calculated using (9) and the electron density in the maps. The histograms that form the basis of (9) are calculated from experimental density for one set of proteins and the correlation is calculated for another. The pair of templates that yields the highest correlation is first identified. Then, one by one, the template that increases this correlation by the largest amount is added to the group, until *N*_final_ templates are chosen.

### 2.7. Indexing the rotations for each template to reduce computational requirements

The slowest step in applying the procedures described here consists of calculating the maximum correlation of local modified density with each of the *N*_final_ templates, considering as many as 158 rotations of each template (or local density) for each point. We have developed a simple indexing system that reduces the number of rotations that need to be considered for each template. The index for a point *x* is based on the density at *M* points near *x* (typically, *M* = 9 and the points are chosen to be approximately uniformly distributed on a sphere of radius 0.9*r*_max_ centered at *x*). Point *m* is given an local index *i*_*m*_ from 0 to 3, based on the local density at that point (ρ ≤ −σ, −σ < ρ ≤ 0, 0 < ρ ≤ σ or ρ > σ), where σ is the r.m.s. of the entire map. An overall index *I* is then calculated for the local density from the relation 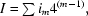
where the sum is over the *M* nearby points. Next, the relationship between the index *I* and the best rotation is tabulated for each of the templates using high-quality experimental maps containing added errors. For each point in each map used above to calculate statistics of the correlation of templates with local modified density, the index *I* is calculated and the optimal rotation is noted for each template. An indexing table is then constructed in which each index *I* is associated with a list of preferred rotations for each template. The table is constructed so that about 95% of the time the optimal rotation for a given template is contained in the list. This indexing procedure reduces the number of rotations that need to be considered by about a factor of five. Other indexing methods could be applied that might further reduce the number of rotations to be considered (*e.g.* Funkhouser *et al.*, 2003 ▶).

### 2.8. Using local patterns to create a new estimate of electron density

The pattern of density near a point *x* in an electron-density map can be analyzed using (8) to produce a probability distribution, *p*(ρ|{CC_obs_}), for the electron density at *x*. The estimate from (9) of density at *x*, ρ_est_ (and the uncertainty in this estimate, σ_est_, if desired), can then be used to construct a new estimate of the electron density in the map. This ‘recovered image’ of the electron-density map can be visualized with or without smoothing, can be used as a target for statistical density modification (Terwilliger, 2000 ▶) or can be combined directly with the original electron-density map to obtain an improved map.

**Figure 1:**
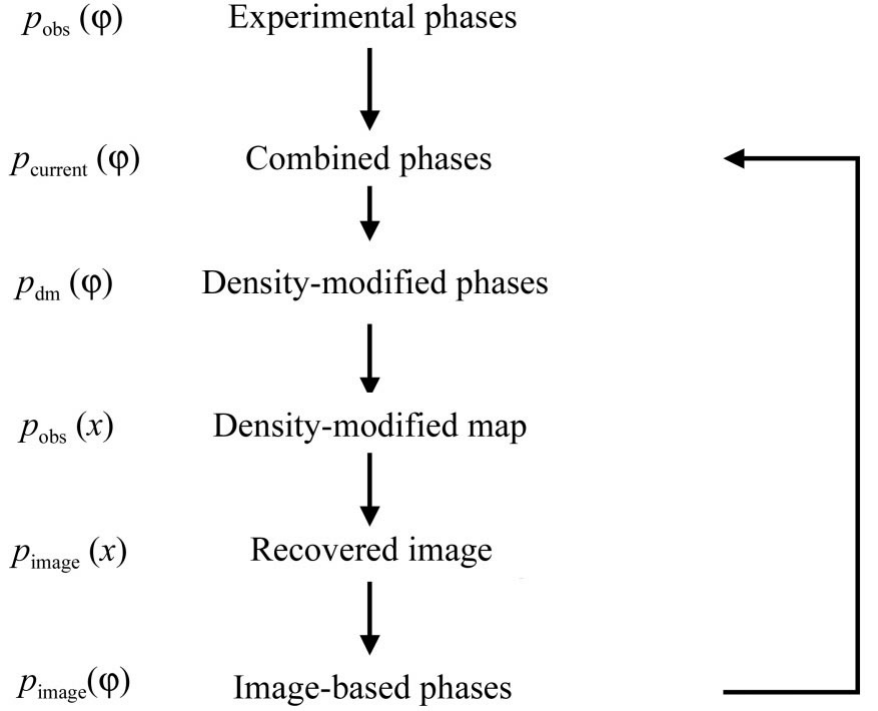
Outline of procedure for density modification using local patterns.

We have used an iterative procedure to combine the information from the recovered image with the information present in an experimental electron density (Fig. 1 ▶). In the first cycle, the starting phase probabilities are experimental values and in all cycles the amplitudes are experimental values. In each cycle, the starting phases and amplitudes are subjected to density modification (*e.g.* statistical density modification or other related methods) to obtain the best possible electron-density map without using any pattern-based information. This density-modified map is then analyzed for local patterns and an image of the map is recovered. Thirdly, the density in the recovered image is used all by itself to estimate phase probabilities. This third step is carried out here using statistical density modification (Terwilliger, 2000 ▶) as described below, but could be performed using σ_*A*_-based methods (Read, 1986 ▶). Finally, the phase probabilities from the recovered image are combined with the original experimental phase probabilities to yield the starting phase probabilities for the next cycle. The process is iterated until changes in the density-modified map from cycle to cycle are small (typically one to five cycles). The density-modified map from the final cycle is then suitable for interpretation.

### 2.9. Using statistical density modification to estimate phases based on a target electron-density function

Statistical density modification (Terwilliger, 2000 ▶) is a procedure for calculating crystallographic phase probabilities based on the agreement of the map resulting from these phases with prior expectations. Any set of prior expectations about the map can be included in this procedure. In particular, if an estimate of electron density is available for all points in the map (*e.g.* the recovered image obtained in the procedure described above), then this estimate can be used as prior information about the map. In this procedure, observed values of the amplitudes of structure factors are used and an estimate of uncertainty in the electron density is required. This procedure is used to estimate phase probabilities from a recovered image, where the expected electron density is simply the best estimate from (9) and the uncertainty is taken to be a constant everywhere given by the r.m.s. of a map calculated with the observed structure-factor amplitudes.

## 3. Results and discussion

### 3.1. Removing information about electron density at *x* from the local electron density

**Figure 2:**
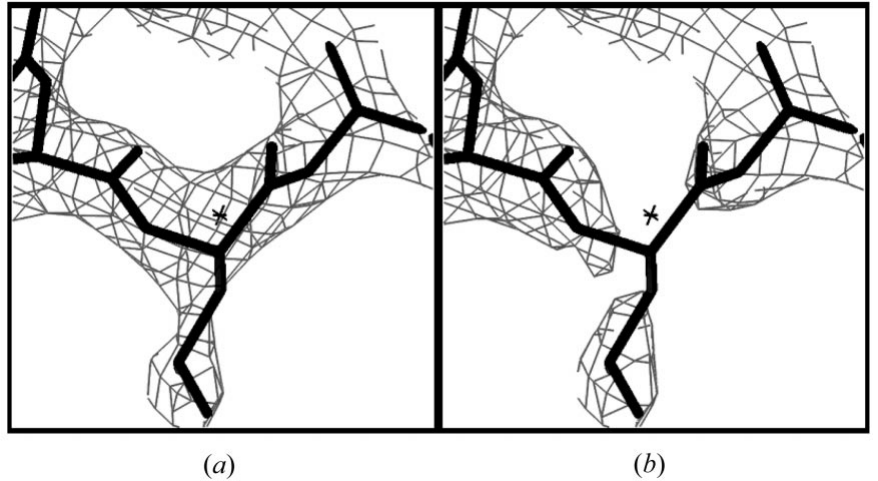
Creating the local modified density function *g*_*x*_(Δ*x*). (*a*) Density in the IF5A electron-density map is shown with contours at 1.5σ. The atomic model used to calculate the map is shown and the central point (‘*x*’) is marked with an asterisk. (*b*) Modified local density *g*_*x*_(Δ*x*) calculated using (5) corresponding to the map in (*a*) is shown. All electron-density maps were created with *MAPMAN* (Kleywegt & Jones, 1996 ▶) and *O* version 8.0 (Jones *et al.*, 1991 ▶).

An important aspect of the pattern-matching density-modification method presented here is that it is designed to yield an estimate of the electron density that has errors uncorrelated with the errors in the original map. This is accomplished by using only information from the region around a point *x* to estimate the density at *x* and not including any information about the density at *x* in the process, as described in §[Sec sec2]2. Fig. 2 ▶ illustrates this process of removing information about electron density at *x*. Fig. 2 ▶(*a*) shows a section of a density-modified MAD electron-density map for initiation factor 5A (IF5A; Peat *et al.*, 1998 ▶) in the region near a particular point *x* (the point *x* is designated by a star at the center of the figure). Note that the density at *x* is positive in this case. In Fig. 2 ▶(*b*), the density is adjusted to remove the information about the density at *x* from *x* and from all neighboring points. This calculation essentially consists of subtracting the origin of a normalized Patterson function corresponding to this map, multiplied by the value of the density at *x* minus the mean local density, from all neighboring points, as described in §[Sec sec2]2. This calculation has the effect of setting the value of the density at *x* to the mean density in the local region, setting the density very near *x* to intermediate values and leaving the value of points far from *x* unchanged.

### 3.2. Common local patterns in protein electron-density maps

The analysis of local patterns in electron-density maps was carried out using the density-modified MAD electron-density map from IF5A, calculated at a resolution of 2.6 Å (PDB code 1bkb; Berman *et al.*, 2000 ▶; Peat *et al.*, 1998 ▶). This was a very clear map with a correlation coefficient to the map calculated from the final refined model of IF5A of 0.82. Local patterns were analyzed for regions centered on each point in this grid, only considering points within 2.5 Å of an atom in the model. Local patterns were identified as described in §[Sec sec2]2 using the modified local density surrounding each point. This approach removes information about the density at *x* from the nearby density. The patterns are selected after considering rotations about the central point, so any rotational differences between templates are not significant in determining their features.

**Figure 3:**
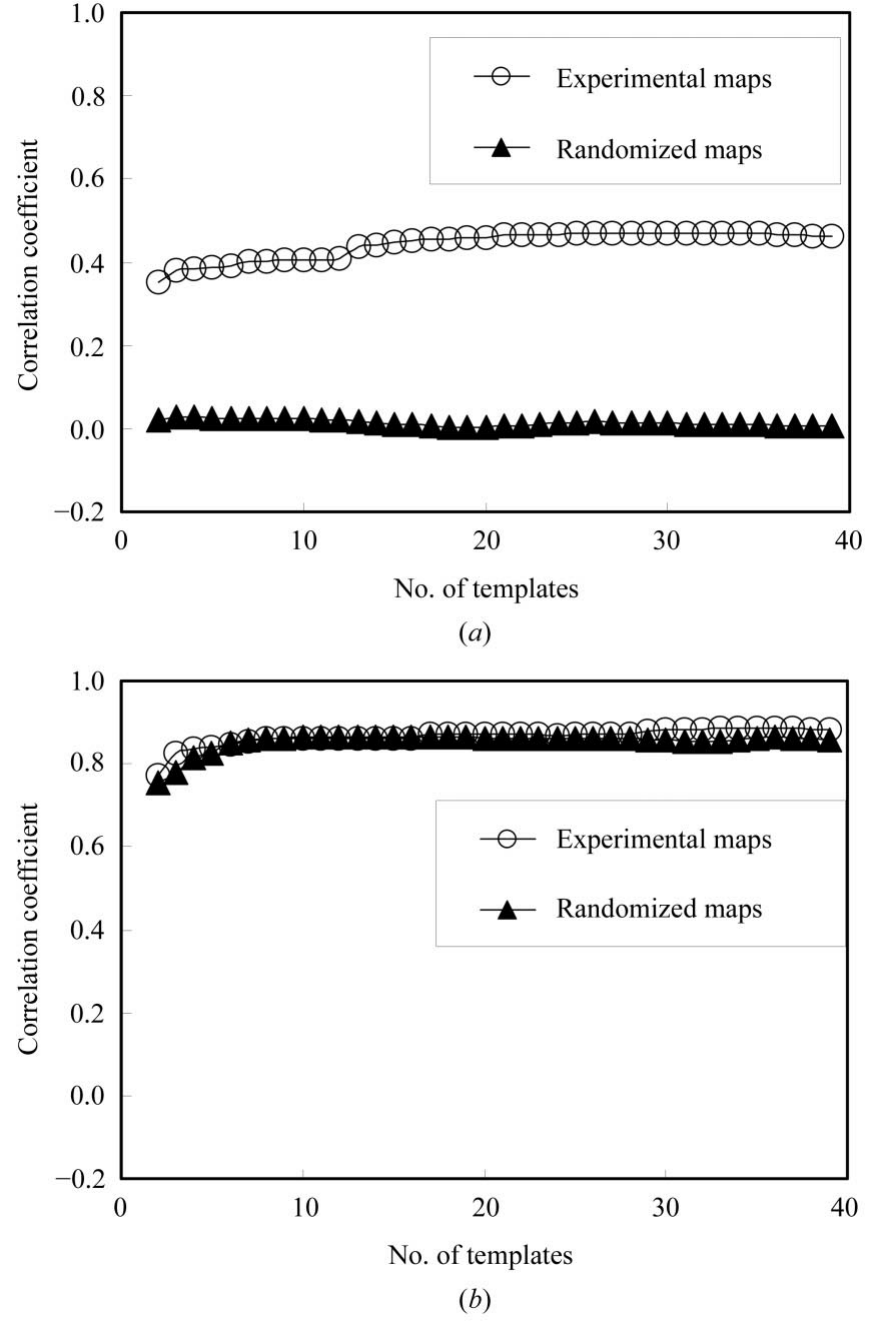
Predictive power of templates. (*a*) Correlation of the recovered density function with true density for the IF5A map (open circles) and for the randomized IF5A map (closed triangles). The correlation of ρ_est_ calculated from (9) with model density ρ is plotted as a function of the number of templates used. For the open circles, the templates were derived from the IF5A map, the histograms from β-catenin and red fluorescent protein maps and the model density and recovered density were from the IF5A map. For the closed triangles, phases were randomized for all three maps before carrying out the calculations. (*b*) As in (*a*), except that the local density was not adjusted to remove information about the density at the central point, so that *g*_*x*_(Δ*x*) = ρ(*x* + Δ*x*).

The final templates were chosen on the basis of their predictive power. The *N*_max_ = 40 templates that were initially created using the model electron-density map for IF5A were then compared with all points in two other density-modified experimental electron-density maps, the armadillo repeat of β-­catenin (Huber *et al.*, 1997 ▶) and red fluorescent protein (Yarbrough *et al.*, 2001 ▶), and correlation coefficients for each template at each point were obtained. The same 40 templates were then compared in the same way with the IF5A map. Finally, subsets of the 40 templates were considered. For each subset of templates, the β-catenin and red fluorescent protein electron-density maps were used to generate histograms and the IF5A map was used to compare the estimates of electron density obtained using (9) with IF5A electron density. In the first cycle of identifying templates, all pairs of templates were considered and the pair yielding the highest correlation was chosen. In subsequent cycles, the additional template that yielded the greatest improvement in correlation was chosen. Fig. 3 ▶(*a*) (open circles) shows the correlation of estimated and model density as a function of the number of templates used. Much of the information is contained in just two templates and almost all the rest is in the first 20. Based on this observation, we have used 20 templates for the remainder of this work.

The fundamental property of macromolecular electron-density maps that is used in our approach is that different local patterns of density in these maps are associated with different values of the density at their central point. The open circles in Fig. 3 ▶(*a*) show that such an association exists and that only a small number of templates are needed to describe it. We next tested whether a similar association exists for random maps. The closed triangles in Fig. 3 ▶(*a*) were obtained in the same way the open circles, except that all the maps were calculated after randomizing all the crystallographic phases. The closed triangles in Fig. 3 ▶(*a*) show that there is essentially no association between local patterns of density and density at their central points for the random maps. This means that the correlations between patterns and densities at their central points is a feature of protein-like maps and not a feature of maps with random phases.

An important part of the present approach was the removal of information about the density at a point *x* in the analysis of the patterns surrounding *x* using (5). The reason for doing this was to obtain an estimate of the density at point *x* that is independent of the current value of density at that point. Fig. 3 ▶(*b*) shows that this choice of methods is also important for discriminating between patterns that arise from noise and those that arise from protein-like features. Fig. 3 ▶(*b*) was calculated in exactly the same way as Fig. 3 ▶(*a*), except that the local density was not adjusted to remove information about the value of the density at the central point and a completely new set of templates and statistics was used, reflecting this different approach. This was accomplished by not applying (5) to the local density. The open circles in Fig. 3 ▶(*b*) show that if the local density is not adjusted to remove information about the central point, then templates can be obtained that give a very high correlation between the value of the density calculated from (9) and the actual density. However, this correlation is likely to be almost entirely due to the fact that information about the central point is included in both the templates and the correlations. Supporting this interpretation, the closed triangles in Fig. 3 ▶(*b*) show that randomized maps give essentially the same correlations as protein electron-density maps when the information about the central point is not removed from the calculations.

**Figure 4:**
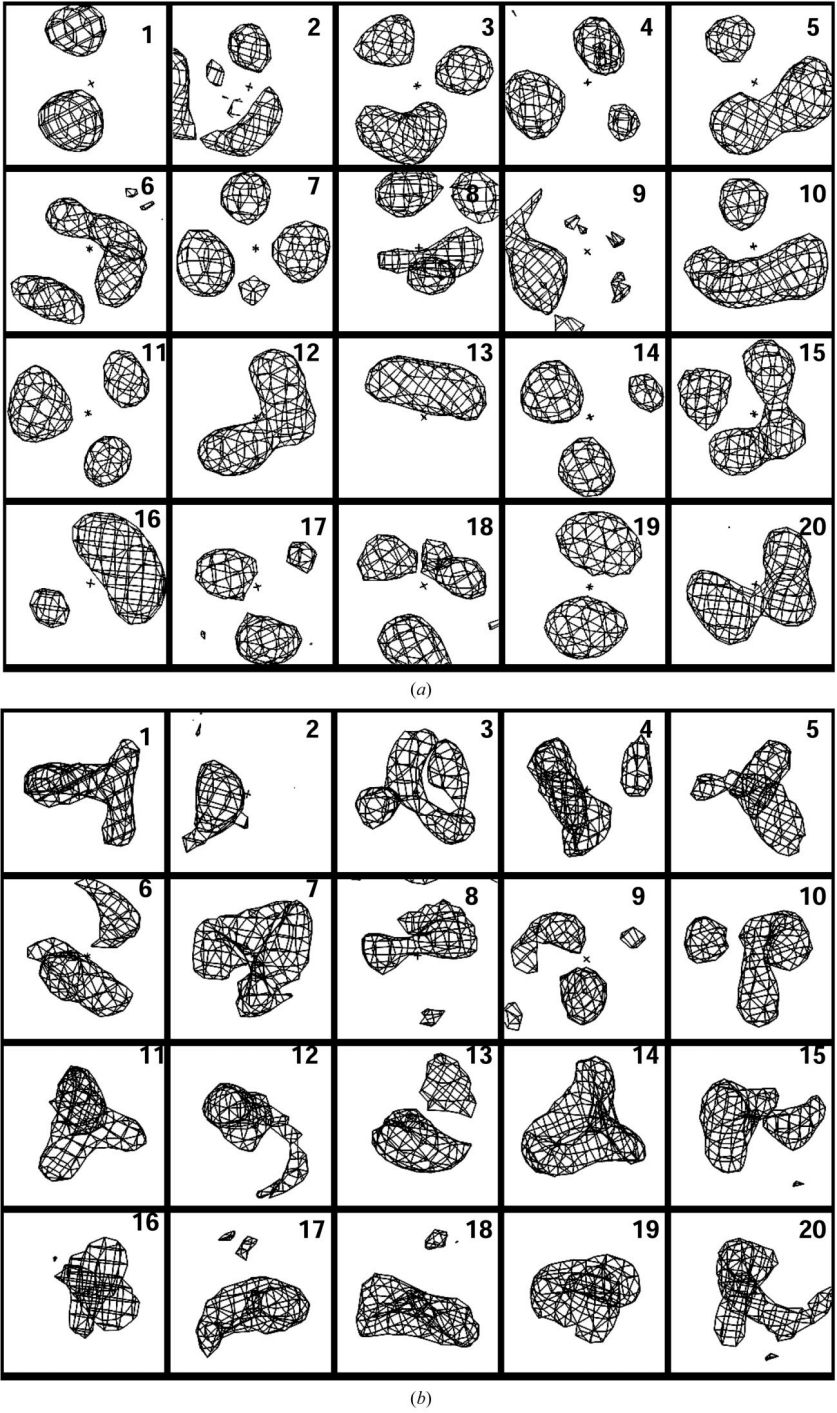
Templates of local density calculated at a resolution of 2.6 Å. The templates are arranged in order of decreasing contribution to the information about the density at the central point. The sections shown are 8 × 8 Å; only the spherical region 4 Å in diameter at the center of each figure is used in the pattern-matching process. Contours at +1.5σ (*a*) and −1.5σ (*b*, templates in the same orientation as in *a*) are shown.

Figs. 4 ▶(*a*) and 4(*b*) show contours of positive density corresponding to the *N*_max_ = 20 templates obtained. The templates are arranged in order of decreasing contribution to the estimates of density. The patterns are very simple, typically containing one to three spherical or extended regions of positive density and one or more rings or regions of negative density in various relations to the central point. Some of the pairs of templates are similar (for example, Nos. 17 and 18) and, as shown in Fig. 3 ▶, the number could be reduced further with just a small reduction in predictive power. The patterns found in some of the templates are related in a simple way to atomic coordinates in the structures used to generate the templates. For example, Fig. 2 ▶ shows the density surrounding a point located near a C^α^ atom, the junction of three chains of atoms. This density, after removing the information about the density right at this point, is most closely similar to pattern No. 12 in Fig. 3 ▶, which consists of a curved lobe of density adjacent to the origin.

The core of the method described here is the association of different templates with different expected values of electron density at the point that is at the center of the templates. The electron density near a point *x* in a map is compared with the 20 templates and the two templates that match the density most closely are identified. The procedure is first performed with high-quality experimental maps to associate pairs of templates with expected density and then with an observed map to estimate the values of electron density in a high-quality version of the observed map. In order to use as much information as possible, the process is carried out in a probabilistic fashion, considering the possibility that any pair of patterns might best match the density in a high-quality version of the observed map.

The 20 patterns are each associated with different average values of density at their central points. For example, template No. 1 contains two spherical regions of positive density situated on opposite sides of the origin. At locations where this pattern is the one that best matches the density in model maps, the mean density at the central point is about −0.3 ± 0.6 (on an arbitrary scale with the mean of the map equal to zero). Template No. 12 contains a curved lobe of positive density immediately adjacent to the origin. Template No. 12 is associated with mean density of about 0.6 ± 0.9. Table 1 ▶ lists the density associated with locations where each of the 20 templates best match the local modified density in model maps.

### 3.3. Reconstructing model electron density using correlations with local patterns

**Figure 5:**
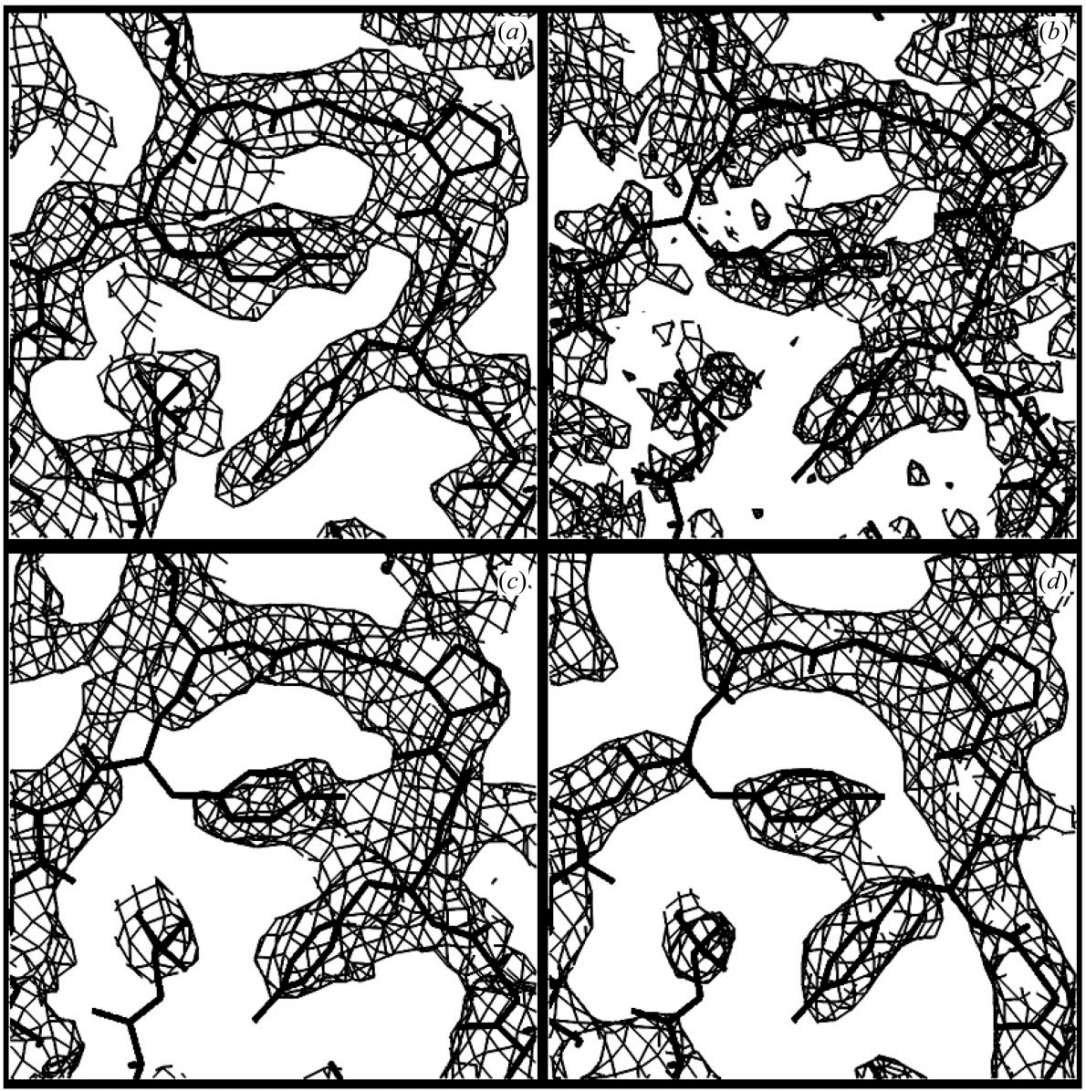
Template matching using model electron density with errors based on the structure of gene 5 protein at a resolution of 2.6 Å. (*a*) Model map with Gaussian phase errors adjusted to yield a correlation to the perfect map of 0.81. (*b*) Estimated electron density reconstructed from the map in (*a*). (*c*) Density in (*b*) after smoothing with a spherical smoothing function with a radius of 1.5 Å. (*d*) Map calculated with model structure-factor amplitudes and with phases estimated using statistical density modification based on the reconstructed density in (*c*). All contours are at 0.8σ.

The templates shown in Fig. 4 ▶ and the density typically associated with them listed in Table 1 ▶ can be used to reconstruct an image of an electron-density map. Fig. 5 ▶ shows an example using model data so that errors can be readily analyzed. Fig. 5 ▶(*a*) shows a section of model electron density with errors calculated using the structure of gene 5 protein (PDB code 1vqb; Skinner *et al.*, 1994 ▶) at a resolution of 2.6 Å. The errors in the phases were adjusted so that the map had a correlation coefficient to the perfect map of 0.81. The estimated electron density reconstructed from this map is shown in Fig. 5 ▶(*b*) and a version of this density, smoothed with a radius of 1.5 Å, is shown in Fig. 5 ▶(*c*). Finally, phases were estimated using statistical density modification based on the model structure-factor amplitudes from the reconstructed density (Fig. 5 ▶ *d*). The reconstructed density has a correlation coefficient to the original (model) map of 0.19, the smoothed image has a correlation of 0.38 and the map calculated with phases obtained from the reconstructed density and model amplitudes has a correlation coefficient of 0.46.

As model data were used to obtain the images in Fig. 5 ▶, it is possible to analyze the errors in the recovered image and determine whether they are in fact independent of the errors in the original map. The errors in electron-density maps are somewhat complicated as they come from errors in phase angles. A simplified error model in which the values of the electron density in two maps *y*_1_(*x*) and *y*_2_(*x*) have correlated errors is assumed for the present analysis. For convenience, in this analysis the maps *y*_1_(*x*), *y*_2_(*x*) are each normalized to an r.m.s. value of unity and a mean of zero. In this error model, each map has a component that is related to *t*(*x*), the true density in a perfect map (also normalized in the same way), each map has a component *c*(*x*) that is an error term unrelated to *t*(*x*) but that is the same in the two maps and each map has an independent error term *e*_1_(*x*) and *e*_2_(*x*). As this is model data, we know the values of *t*(*x*) as well as the values of *y*_1_(*x*) and *y*_2_(*x*), 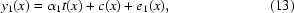

               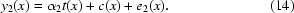
In this model case, the coefficients α_1_ and α_1_ can be estimated from the known maps *t*(*x*), *y*_1_(*x*) and *y*_2_(*x*), 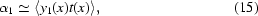

               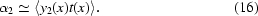
We can then estimate the correlation of errors CC_errors_ with the relation
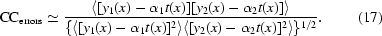
Using (17), we find that the correlation coefficient of the errors in the starting map with errors with the errors in the recovered map in Fig. 5 ▶(*b*) is −0.01. The same calculation for the recovered smoothed map in Fig. 5 ▶(*c*) leads to a correlation coefficient of the errors of −0.02. Similarly, the calculation for the map in Fig. 5 ▶(*d*) obtained using phases calculated from the recovered image and model amplitudes lead to a correlation of errors of −0.04. This indicates that the errors in the recovered image are not correlated with the errors in the original map.

We have found that the independence of errors is not as perfect when density-modified phases are used. To examine this, we started with model phases and amplitudes, introduced errors into the phases, leading to an electron-density map with a correlation to the perfect map of 0.6, and then carried out statistical density modification on this map (not including any local pattern information), leading to a density-modified map with a correlation to the perfect map of 0.83. This density-modified map was then analyzed for local patterns as described above. In this case the smoothed recovered image had a correlation to the perfect map of 0.50. The correlation of errors with the density-modified map was 0.21, considerably higher than in the case where the map used for pattern identification had completely random errors. This suggests that the method might not be quite as effective when used on density-modified maps as on experimental maps.

### 3.4. Reconstructing electron density from density-modified experimental maps using correlations with local patterns

**Figure 6:**
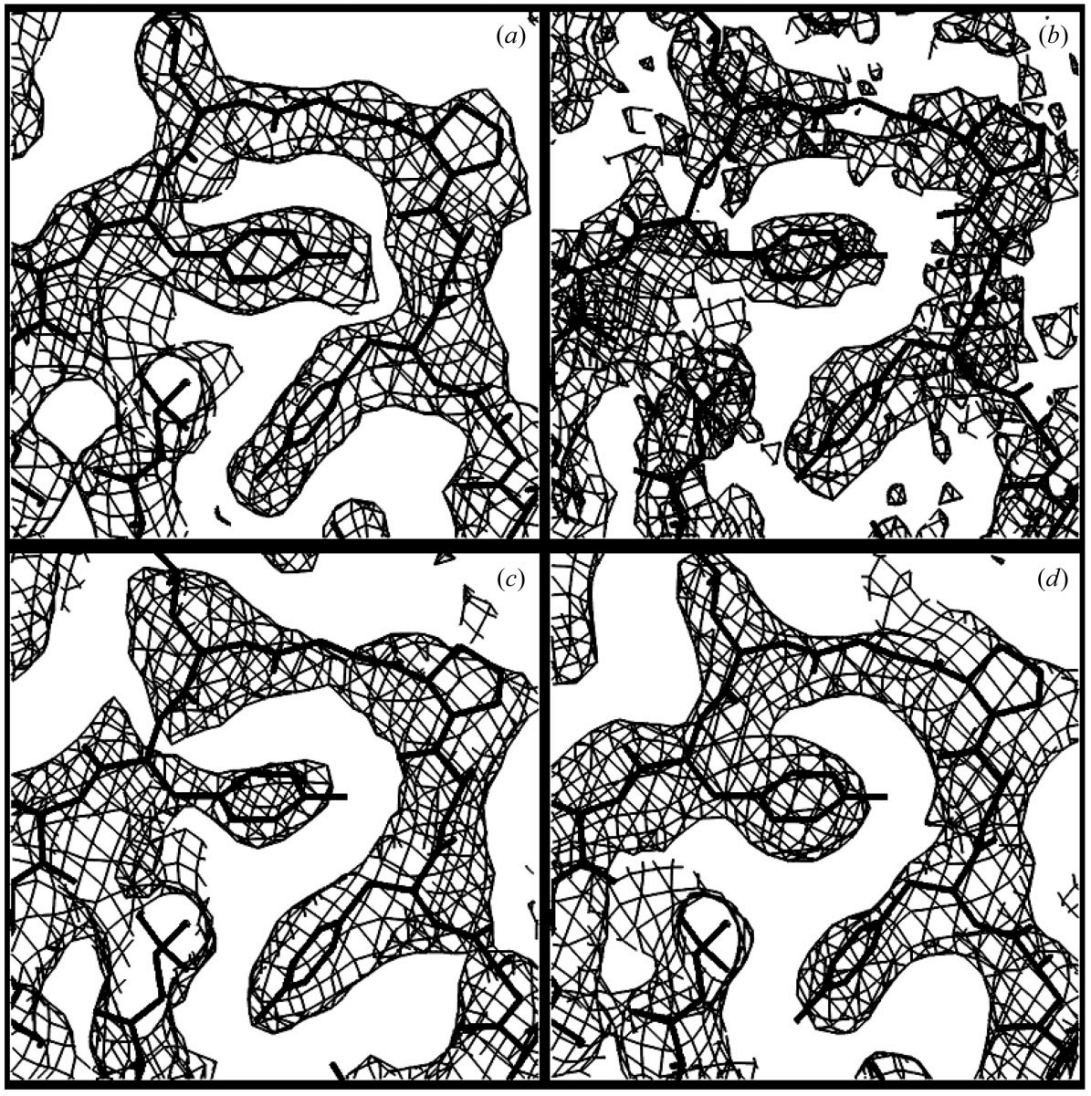
Template-matching using gene 5 protein MAD data. As in Fig. 5 ▶, but using experimental MAD data instead of model data.

The analysis described above was carried out with electron density calculated from models so that the error analysis could be performed in detail. We next applied the method to electron density obtained from a MAD experiment so that its utility with real data could be examined. The electron density obtained after applying statistical density modification (Terwilliger, 2000 ▶) to three-wavelength MAD data from gene 5 protein (PDB code 1vqb; Skinner *et al.*, 1994 ▶) was used as the starting point for this analysis. This *RESOLVE* electron-density map had a correlation coefficient of 0.79 to the model density calculated from PDB entry 1vqb. Fig. 6 ▶(*a*) shows a section through this density-modified map. Local pattern analysis was applied to this map as described above. Fig. 6 ▶(*b*) shows the image that was recovered from this map, Fig. 6 ▶(*c*) shows a smoothed version of this image and Fig. 6 ▶(*d*) shows the map obtained using phases calculated from the recovered image and observed structure-factor amplitudes. The recovered image in Fig. 6 ▶(*b*) has a correlation of 0.25, the smoothed recovered image in Fig. 6 ▶(*c*) has a correlation of 0.42 and the map calculated using phases from the recovered image in Fig. 6 ▶(*d*) has a correlation of 0.52.

An approximate version of the error analysis described in the previous section for Fig. 4 ▶ was carried out for the maps in Fig. 6 ▶. In this analysis, the ‘true’ density was taken to be the density calculated from the model of gene 5 protein (PDB code 1vqb). The correlation of errors between the starting *RESOLVE* map in Fig. 6 ▶(*a*) with the errors in the recovered image in Fig. 6 ▶(*b*) was 0.15 and the correlation of errors between the starting *RESOLVE* map with the errors in the smoothed recovered image in Fig. 6 ▶(*c*) was 0.23. The correlation of errors in the map calculated using phases from the recovered image in Fig. 6 ▶(*d*) with the errors in the starting *RESOLVE* map was 0.36. This means that the errors are not highly correlated in this analysis, but that they are also not completely independent. Part of the correlation of ‘errors’ could be because of the fact that the ‘true’ density is not known and the errors are estimated using model density for gene 5 protein. Consequently, any errors in this model density would lead to correlation of ‘errors’ in all the maps in this analysis.

### 3.5. Combination of phase information from local pattern identification with experimental phase information

**Figure 7:**
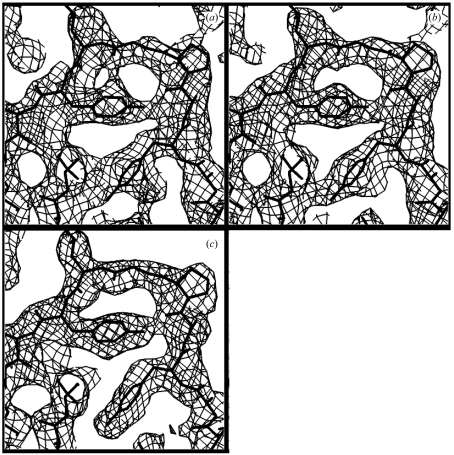
Phase improvement using template matching on gene 5 protein MAD data. (*a*) *SOLVE* electron-density map for gene 5 protein. (*b*) Electron-density map calculated using observed structure-factor amplitudes and combined phases. The combined phases consisted of the *SOLVE* phase estimates combined with the phases estimated using statistical density modification based on the reconstructed density shown in Fig. 6 ▶(*b*). (*c*) *RESOLVE* electron-density map after one cycle of statistical density modification starting with the map shown in (*b*). All contours are at 0.8σ.

Fig. 6 ▶(*d*) shows an electron-density map calculated using observed structure-factor amplitudes for gene 5 protein and phase probabilities obtained using statistical density modification on the reconstructed image in Fig. 6 ▶(*b*). These phase probabilities were then combined with the original phase probabilities from the three-wavelength MAD experiment to yield a set of phase probabilities and a new electron-density map. The original *SOLVE* electron-density map (Terwilliger & Berendzen, 1999 ▶) using experimental phases is shown in Fig. 7 ▶(*a*). This map has a correlation with the model gene 5 protein map of 0.56. The electron-density map calculated from combined phases is shown in Fig. 7 ▶(*b*). This new electron-density map has a correlation to the model map of 0.65. Finally, the combined phases and the experimental structure-factor amplitudes were used in statistical density modification using the same parameters as those used to obtain the original *RESOLVE* phase probabilities. The resulting map is shown in Fig. 7 ▶(*c*); it is very similar to the original *RESOLVE* map shown in Fig. 5 ▶(*a*), but is slightly improved, with a correlation to the model gene 5 protein map of 0.82 (compared with 0.79 for the original *RESOLVE* map).

A key element of the process used here is to remove information about the density at each point *x* from the analysis of patterns of density around of *x*. We tested the importance of this step by repeating the entire process of generating templates and histograms and then applying them to the gene 5 protein MAD data, but without removing this information. In this case, the recovered image had a higher correlation with the model map than in the test case described above (0.55 compared with 0.25) and the smoothed recovered image had a correlation of 0.59, compared with 0.42. On the other hand, the correlation of errors between the recovered image and the starting *RESOLVE* map was also much higher (0.68 compared with 0.15), as was the correlation of errors between the smoothed recovered image and the starting *RESOLVE* map (0.85 compared with 0.23). Finally, the resulting combined phases were used as a starting point for density modification, but in this case no improvement in the final map was obtained (correlation coefficient with the model map of 0.79 in both cases), supporting the idea that this step is an important element in the process.

### 3.6. Iterative local pattern identification and density modification

**Figure 8:**
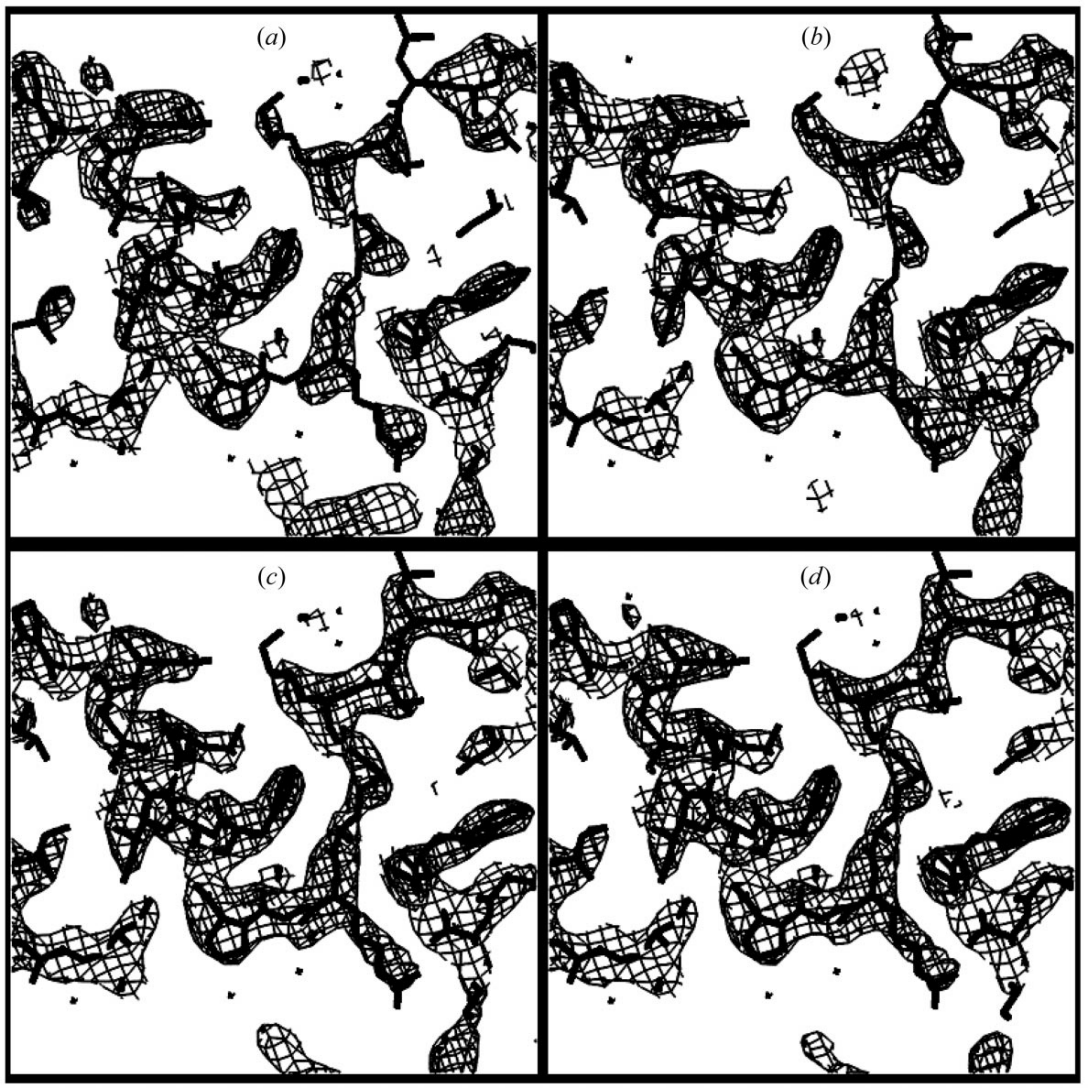
Phase improvement using template matching on nusA SAD data. (*a*) *RESOLVE* electron-density map for nusA protein calculated without pattern matching. (*b*), (*c*) and (*d*) Electron-density maps after one, three and five cycles of density modification including pattern matching, respectively. All contours are at 1.5σ.

Fig. 1 ▶ illustrated an iterative process for phase improvement based on the local pattern identification described here. In this process, the pattern-identification step is always carried out on the best available map and then the resulting phase information is combined with experimental phase information to yield an improved starting point for density modification. The first cycle in this iterative process for phase improvement is identical to the process described above. Subsequent cycles simply iterate the process. Fig. 8 ▶ shows the results of applying the process to SAD data collected on nusA protein from *Thermotoga maritima* (D. H. Shin, H. T. Nguyen, J. Jancarik, H. Yokota, R. Kim & S.-H. Kim, unpublished data; PDB code 1l2f) at a resolution of 2.4 Å. Fig. 8 ▶(*a*) shows a section through the *RESOLVE* electron-density map obtained without using local pattern matching. Figs. 8 ▶(*a*), 8 ▶(*b*) and 8 ▶(*c*) show the density-modified map after one, three and five cycles using local pattern matching. The correlation coefficient of the starting *RESOLVE* electron-density map with a map calculated from the refined model of nusA is 0.65; the map after five cycles has a correlation of 0.85.

Table 2 ▶ summarizes the results of applying this process to experimental data from crystals of several different proteins. The greatest improvement in map quality was obtained for cases where the original *RESOLVE* map had a correlation with the model map of less than 0.7, with smaller improvements obtained when the *RESOLVE* map was better than this. To provide a rough measure of the utility of the method, the automatic model-building capability of *RESOLVE* was applied to the maps obtained for each structure with and without information from local patterns (Table 2 ▶). The percentage of main-chain residues built was essentially the same with and without information from local patterns for all the structures except nusA, which increased from 49 to 56% with the use of local patterns. On the other hand, the percentage of residues assigned to sequence and side chains built increased, on average, from 11 to 24% for those structures where the map correlation was considerably improved (UTP-synthase, nusA, NDP-kinase). This indicates that the map improvement can be enough to make a significant difference in the ability of automated procedures to build a complete atomic model.

Although the templates used in this procedure were calculated using data to 2.6 Å, the procedure is not strongly dependent on resolution. Using the nusA data as a test case, the effect of resolution was examined by truncating the analysis at resolutions of 2.4 (all data), 2.6, 2.8 and 3.0 Å, respectively. The correlation of the original *RESOLVE* maps at each of these resolutions with the model maps calculated at the same resolutions were similar (0.65, 0.66, 0.69 and 0.69, respectively), as were the correlations of the final maps density modified including the local pattern information (0.85, 0.85, 0.85 and 0.86, respectively).

## 4. Prospects

We have shown here that local features of electron-density maps can be used as an important source of information in a density-modification procedure. The improvements in map quality obtained using the information from local patterns range from none (0.87 to 0.87 for β-catenin) to small (from 0.79 to 0.82 in correlation coefficient for gene 5 protein) to very substantial (from 0.65 to 0.85 in correlation coefficient for nusA).

The computational requirements of the methods are moderate. Carrying out a complete set of five cycles of pattern identification and density modification using local patterns takes 90 min on a Compaq 833 Mhz Alpha for the ‘hypothetical’ protein from *P. aerophilum* listed in Table 2 ▶ (494 amino acids); standard density modification without using local pattern information takes about 5 min. Memory requirements are moderate as well: the libraries of patterns and indexing tables are large and (along with other parts of the software) require approximately 700 MB of swap space or more.

There are many additional applications of the procedures that we have developed here. A key aspect of the methods is that the image that is recovered from an electron-density map has errors that are relatively uncorrelated with those in the original map. This allows the use of the recovered image in phase improvement in the moderate-resolution range demonstrated here. It is also possible that the same approaches could be used for low-resolution as well as very high resolution phasing and phase extension. Additionally, the independence of errors means that an image recovered from a random map will have little or no correlation to the original map, while an image recovered from a map that has protein-like features will have a correlation. Consequently, the method could be used to evaluate the quality of protein electron-density maps. Similarly, points that are in the solvent region of a crystal will have local features unlike those found in the protein region and the methods described here could be used to distinguish the protein from solvent regions.

A weakness of the pattern-matching approach developed here is that it cannot readily distinguish protein-like features that are the result of systematic bias or errors in a map from those that actually reflect protein structure. This may be reflected in the small but significant correlation of errors between the density-modified model gene 5 protein map and its recovered image described above. Perhaps more importantly, it means that the method in its present form is not as well suited to improving maps that contain significant bias towards protein-like patterns of density, such as those obtained using phases from an atomic model, as it is to improving maps in which the errors are essentially random, such as those obtained by experiment.

A useful extension of the methods described here will be to recalculate the templates and histograms using data in various resolution ranges and using various radii for the regions considered in obtaining templates and to apply the appropriate set to experimental data. The effects of the grid spacing used in calculations could also be investigated. The use of correlations to more than two templates could be used in (8) in estimates of local density (although our preliminary investigations indicated that using a third template added very little information to the calculation). In each of the cases described here, the templates and histograms were obtained from model maps calculated at a resolution of 2.6 Å. The use of templates at varying resolutions could potentially increase the applicability of the method to a wider resolution range. Other extensions include examining the patterns in different classes of protein structures and in crystals that contain other structures such as nucleic acids or various ligands.

## Figures and Tables

**Table 1:** Templates of local electron density calculated at a resolution of 2.6 Å

Template	Mean density at center (arbitrary units, with mean of map equal to zero)	Variance of mean density
1	−0.29	0.60
2	0.06	0.73
3	−0.63	0.59
4	−0.55	0.60
5	−0.38	0.81
6	0.49	0.95
7	−0.68	0.56
8	−0.05	0.72
9	−0.40	0.55
10	−0.32	0.70
11	−0.41	0.74
12	0.62	0.87
13	0.37	0.72
14	−0.46	0.66
15	0.46	1.00
16	−0.17	0.76
17	−0.03	0.78
18	−0.15	0.66
19	−0.27	0.81
20	0.49	1.00

**Table 2:** Application of iterative statistical density modification with local pattern recognition For each experimental data set, density modification was carried out using default inputs for *RESOLVE* (Terwilliger, 2000) and phase probabilities calculated using *SOLVE* (Terwilliger & Berendzen, 1999). The process shown in Fig. 1 ▶ was then carried out, including the identification and use of local patterns of density. Non-crystallographic symmetry was not included in any density-modification procedures in these tests. The correlation coefficient of the resulting electron-density maps to those calculated with phases obtained from the refined models of each structure are listed. Additionally, the number of residues that could be automatically modeled and assigned to sequence and the number that could be modeled (whether or not assigned to sequence) with *RESOLVE* (Terwilliger, 2003*a* ▶,*b* ▶) using default parameters are listed. As the number of residues obtained with automated model building is somewhat sensitive to the parameters and details of the methods used, models were built with versions 2.02, 2.03, 2.04 and 2.05 of *RESOLVE* and the average numbers of residues built are reported.

Structure	UTP-synthase†	Armadillo repeat of β-catenin‡	Gene 5 protein§	Hypothetical (*P. aerophilum* ORF)¶	NusA††	NDP-kinase‡‡
Resolution (Å)	2.8	2.7	2.6	2.6	2.4	2.4
Type of experiment	SAD	MAD	MAD	MAD	SAD	MAD
*RESOLVE* map correlation to model map						
With local patterns	0.760	0.874	0.815	0.821	0.847	0.649
Without local patterns	0.727	0.872	0.786	0.811	0.648	0.586
Residues in refined model	1012 (2 × 506)	455	86	494 (2 × 247)	344	556 (3 × 186)
Main-chain residues built by *RESOLVE* (%)						
With local patterns	72	78	72	76	56	76
Without local patterns	72	78	69	76	49	76
Side-chain residues built by *RESOLVE* (%)						
With local patterns	34	58	52	65	21	18
Without local patterns	24	58	51	61	5	4

† Gordon *et al.* (2001 ▶).

‡ Huber *et al.* (1997 ▶).

§ Skinner *et al.* (1994 ▶).

¶ NCBI accession No. AAL64711; Fitz-Gibbon *et al.* (2002 ▶).

†† D. H. Shin, H. T. Nguyen, J. Jancarik, H. Yokota, R. Kim & S.-H. Kim, unpublished work; PDB code 1l2f.

‡‡ Pédelacq *et al.* (2002 ▶).
